# Community nursing needs more silver surfers: a questionnaire survey of primary care nurses' use of information technology

**DOI:** 10.1186/1472-6955-3-4

**Published:** 2004-10-07

**Authors:** Tom Chan, Sarah Brew, Simon de Lusignan

**Affiliations:** 1 Kent Surrey and Sussex Primary Care Research Network (KSSnet.) Surrey and Hampshire Borders Community Trust Camberley, Surrey, UK; 2 Kent Surrey and Sussex Primary Care Research Network (KSSnet.) Three Bridges Practice, Crawley, West Sussex, UK; 3 Senior Lecturer – Primary Care Informatics Department of Community Health Sciences St. George's Hospital Medical School LONDON SW17 ORE UK

## Abstract

**Background:**

In the UK the health service is investing more than ever before in information technology (IT) and primary care nurses will have to work with computers. Information about patients will be almost exclusively held in electronic patient records; and much of the information about best practice is most readily accessible via computer terminals.

**Objective:**

To examine the influence of age and nursing profession on the level of computer use.

**Methods:**

A questionnaire was developed to examine: access, training received, confidence and use of IT. The survey was carried out in a Sussex Primary Care Trust, in the UK.

**Results:**

The questionnaire was sent to 109 nurses with a 64% response rate. Most primary care nurses (89%) use their computer regularly at work: 100% of practice nurses daily, compared with 60% of district nurses and 59% of health visitors (p < 0.01). Access to IT was not significantly different between different age groups; but 91% of practice nurses had their own computer while many district nurses and health visitors had to share (p < 0.01). Nurses over 50 had received more training that their younger colleagues (p < 0.01); yet despite this, they lacked confidence and used computers less (p < 0.001). 96% of practice nurses were confident at in using computerised medical records, compared with 53% of district nurses and 44% of health visitors (p < 0.01.) One-to-one training and workshops were the preferred formats for training, with Internet based learning and printed manuals the least popular (p < 0.001).

**Conclusions:**

Using computers in the surgery has become the norm for primary care nurses. However, nurses over 50, working out in the community, lack the confidence and skill of their younger and practice based colleagues.

## Background

In the UK its National Health Service (NHS) is investing in computerisation. Large contracts have been set that will run until 2013 to provide electronic patient records [1]. Over this timeframe, with the exception of patient-held records, written medical records will largely be phased out, and replaced by the computerised. Instead of each hospital, or other NHS service that a patient attends creating its own record in isolation separate records will be linked via an "electronic spine." To help integration of records the whole NHS is due to move to a single coding system. Currently most of general practice uses Read codes to record structured data in computers [2,3]. There are many other systems currently in use in the other parts of the health service, but this is all due to come to an end with the whole NHS moving to use a classification system called SNOMED CT (Systematized Nomenclature of Medicine – Clinical Terms [4,5]) In addition, computer use will be become more and more necessary, as ever increasing amounts of information to support learning and the delivery of high quality care, are placed on-line [6,7]. Every general practice premises has a connection to NHSnet and via this the Internet, to facilitate access [8].

In this environment primary care professionals will need the skills to read and enter data in computerised medical records, access guidelines and other information on-line, receive alerts about changes in practice via email and use computer as a learning resource. Nurses: practice nurse, district nurses, health visitors, and others, make up a substantial proportion of the primary care workforce. Previous studies have suggested that they have limited access and low levels of confidence and competence in using IT (Information Technology) [9-11]. However, the data reported in these publications is at least two years old. We therefore conducted this study to discover the extent to which primary care nurses had access to, and were using IT; and to examine whether any particular groups were being left behind.

## Methods

A literature review was carried out of standard bibliographic data bases: CINAHL, British Nursing Index and Medline to identify any recent publications about use of IT or computerised medical record by practice or district nurses and health visitors. In addition the primary care electronic library (PCEL) [12], and information departments in the Community Practitioners and Health Visitors Association (CPHVA) [13] and Royal College of Nursing (RCN) [14] were contacted by email. We did not find any contemporary reports of UK primary care nurse use of IT.

The study took place in a single Primary Care Trust in Sussex, a mixed urban and rural county in southeast England. A Primary Care Trust is a geographically defined locality of approximately 1 – 200,000 people that commissions hospitals, general practitioners and other health providers to deliver comprehensive local NHS healthcare. A PCT can also provide care itself. Most community nurses are employed directly by their PCT, and this PCT is no exception.

All the practices in this PCT are computerised with computers linked to the patient database located in all consulting and treatment rooms; and most offices. In common with nearly all UK general practice the practice is networked and the network is connected to the internet via a fixed ISDN-2 (Integrated Services Digital Network) link. The ISDN (64 K link) actually connects the practices to the NHS own intranet (NHSnet) and via secure gateways to the internet [8]. The NHSnet connection also provides primary care professionals email, as part of this service. In theory there should not be problems with access to IT in NHS primary care.

**Figure 1:**
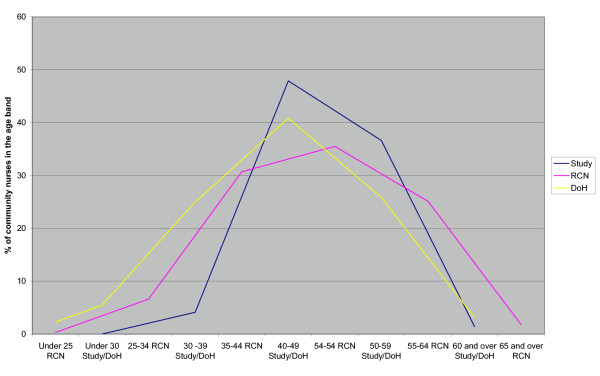
Comparison of the age distribution of the study population with the Royal College of Nursing community nurse members in the southeast of England.

This PCT has a predominantly female (97%) workforce with one-and-a-half times as many nurses in the over 40 age group compared with the national average for nursing [15]. The Nursing and Midwifery Council does not keep separate age-sex details for community nurses, however some information was available from the RCN. The RCN provided information about the age range of community nurses in southeast England. These, although collected using different age bands, show a similar population distribution as that found in this sample. The department of health produces details of community nurses by age band [16]. Although, these data do not include practice nurses, and are a national sample, they also show a similar distribution. The proportions by age group are shown in Figure 1.

A questionnaire was considered the most appropriate way to gather the information required. Two previous studies indicated that primary care nurses appeared willing to complete the questionnaires and provide rich data [10,11]. The design consisted of questions which required "tick box" answers, or rating choices on a simple four point scale. It had 12 questions spread over three sides of A4 with a fourth side available for comments; it could be completed in less than five minutes. A copy of the questionnaire is attached as an appendix to this paper [see Additional file 1], or can be downloaded from: .

The questions were based on those used in two earlier studies; with the clarity of questions was improved. Especially distinguishing between a clinical computer system (i.e. one that is used to enter data into an electronic patient record) from other "work" computer systems; and, between home and work use. The questionnaire was tested by a pilot group of nurses located in another PCT in a neighbouring county. The pilot group of six nurses were asked to complete the questionnaire on their own. They were then invited to join a discussion group – where the purpose of the study was explained. Then the questions were gone through one by one to identify any ambiguity. The nurses were also invited to suggested alternative or additional questions. They were given SB's contact details should anything come to mind later.

Ethical approval was obtained from the local research ethics committee to conduct the study.

Primary care nurses were identified using a list provided by the PCT. The questionnaire was sent out with a covering letter explaining the nature of the study. A courier addressed envelope was provided so the questionnaire could be returned without cost. A reminder and second questionnaire was sent out to non-responders.

The questionnaire responses were coded and entered into SPSS (Statistical Package for Social Sciences) for analysis. The results were analysed by age bands and professional grouping as we wished to test whether either of these factors predetermined the level of computer use. Additional comments were entered into Microsoft Word, and the SPSS data from the questionnaire was placed alongside the relevant comments – they were flagged with the professional group and age group of their originator. Microsoft Excel was used to allow them to be sorted by age group and profession.

**Figure 2:**
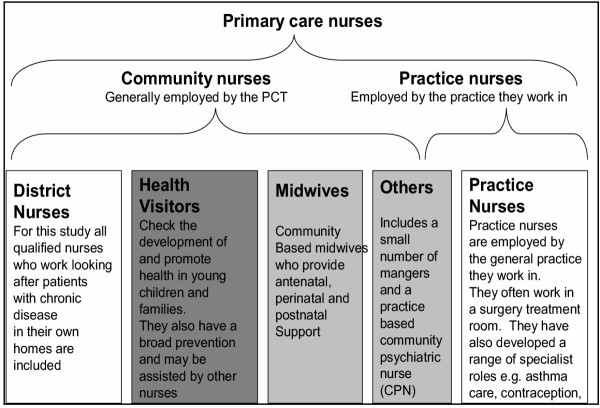
Typology of UK primary care nurses

To simplify the analysis only three age-groups were considered: age under 30, 30 to 39, 40 to 49, and 50 and over. There were no nurses under 30, and only one over 60. The nursing professions were divided into: District nurses, health visitors and midwives, practice nurses, and others. In this study qualified nurses who work in the community primarily supporting district nurses or health visitors were analysed as part of that nursing specialist group. The health visitors and community midwives were combined into the "health visitors" group. The term "primary care nurse" is used to embrace all qualified nurses included in this survey; "community nurse" refers to nurses who still do a proportion of their work in patients' homes, Figure 2.

## Results

There was a 67% (67/105) response rate to the questionnaires. There was some variation between nursing groups: the response from practice nurses was 73% (n = 22/30), for district nurses 51% (n = 17/33), for health visitors and midwives 67% (n = 19/28), and other nurses, community psychiatric nurses and managers 64% (9/14).

Computers were readily available to primary care nurses. Over 90% of nurses had access to a personal computer (PC) at work, though for nearly half (45%) this was shared access; home access was even higher – 96%, with very few (12%) describing their access as shared. The remainder had access elsewhere: library, college etc. Not all the computers were connected to the Internet: 11% of those provided a PC at work and 6% at home could not go on line. After taking into account "other" access to the Internet only 3% of primary care nurses lacked access.

The pattern of access to computers, the Internet, and receiving training changed with age. The younger nurses had more access to PCs and the Internet at work, while older nurses had higher levels of access at home, and had received more training. These trends are shown in Table 1; only the receipt of more training in nurses 50 and over was statistically significant (Chi-squared test.) There were more striking differences between the different nursing professions. 90.1% of practice nurses had their own computer at work, with the balance of 9.1% having shared access – three-quarters (76.2%) had Internet access via this computer. By way of contrast their community based colleagues had significantly less access – only 56% of district nurses and 17% of health visitors had their own computer. Most had shared access and a few, 6.3% and 8.7% respectively, no access at all (p < 0.001).

**Table 1:** Trends in access to computers, the Internet and receipt of training with age

	**Age 30–49**	**Age 50 & over**	**All**	**Pearson X^2 ^*p*=**
Has access to own PC when working	59.1%	37.0%	50.7%	0.074
Has own access to Internet when working	47.7%	29.6%	40.8%	0.225
Has Access to PC at home	81.8%	85.2%	83.1%	0.656
Has Access to Internet at home	77.3%	77.8%	77.5%	0.999
Have received computer training	50%	81.5%	62.0	0.008
(n=)	44	27	71	

62% of nurses had received some training, although it was often superficial and run in house to provide basic knowledge of how to use the clinical computer system. Some learned from family members, colleagues or were self-taught, in the use of Microsoft Word and Excel. A few respondents received more formal training on the use of Internet for literature searching from a library, evening classes or as part of high education. Open question data shows that the training received are very diverse both in subjects and level of skills:

"...two fifteen minute sessions in 1991 when we first got computers, a second when system was updated"

"...only one day. All other courses since have been at difficult times or locations"

"...very basic"

"...not sufficient though"

"...no formal courses, only shown by colleague when I was new in post"

"Protected time for PC training would be most valuable – so often this is hurriedly given during the odd 5-minute break rather than in a training session."

"...adequate training is really necessary and protected time; 10–15 minutes between patients is not adequate."

Respondents were asked to rate their preferred format for ICT training on a 4-point scale: from 0 for least desirable to 3 for highly desirable. The result showed that 'one-to-one' training and workshops were the preferred formats for training, printed manual the least (Table 2.)

**Table 2:** Preferred format for training

**Training format**	**Mean rank**
Printed manual	1.73
Tutorial on the Internet	2.68
Lectures	2.46
Workshops	3.75
One to one	4.38

(Friedman Test: n = 42; X^2 ^= 87.01; df = 4; p < 0.001).

The preferred location for the training reflected the format of training requested (Table 3.) Most wanted training to take place one-to-one in their workplace with those wanting workshops looking to see them held in an education or teaching centre. No age or professional group differences were found for the format or venue of ICT training required.

**Table 3:** Preferred location of IT training.

	*% of responses*	*% of cases*
At work	63.7	84.1
In education/teaching centre	29.7	39.1
Library	2.2	2.9
Home (self directed learning)	4.4	5.8
	100%	131.9%

(Multi-response analysis: 3 missing cases; 69 valid cases)

Confidence with computers appeared to be age related, with younger nurses having higher levels of confidence across all the areas of competence than their more senior colleagues. However, only two of these trends, use of spread sheets and electronic patient records (EPR) were found to be statistically significant, see Table 4; though the latter is critically important for patient care. Practice nurses had significantly higher levels of confidence in working with the EPR. 95.5% of practice nurses felt confident compared with 53% of district nurses and 44% of health visitors (p < 0.01).

**Table 4:** Confidence in the use of IT among primary care nurses

	**Age 30–39**	**Age 39–49**	**Age 50 & over**	**X**^2^
Mouse	97.7%	96.3%	97.2%	0.724
Key board	93.3%	81.5%	88.7%	0.291
Word processor	70.5%	51.9%	63.4%	0.128
Spread sheet	43.2%	25.9%	36.6%	0.095
e-mail	79.5%	66.7%	74.6%	0.243
Internet	81.8%	70.4%	77.5%	0.457
Electronic library	52.3%	33.3%	45.1%	0.292
Electronic medical records	79.5%	37.0%	63.4%	*P *= 0.001
n=	44	27	71	

The raw data also suggested that there was a discernable trend in computer usage with age. 90% of nurses age 30 to 39 years used their workplace computer daily, 70% of those 40 to 49, and only 59.3% of those over 50. This trend was not significant, see Table 5.

**Table 5:** Use of computers at work for different age groups of community nurses

	**Age 30–49**	**Age 50 & over**	**Total**
At Least daily	75.0	59.3	69 (n = 49)
At least weekly	13.6	25.9	18.3 (n = 13)
At least monthly or never	11.4	14.8	12.7 (n = 9)
n=	44	27	71

Colleagues were the most used source of information across all ages and nursing professions. Older nurses tended to use books and journals from their personal collections, and to use libraries. However, the use of libraries, books and journals is quite low.

Younger nurses seem to use journals at work or electronic resources more. Neither of these trends, shown in Table 6, were statistically significant. A larger proportion of practice nurses were more confident about using electronic libraries (63%), compared with health visitors (35%), and district nurses (24%); this trend was also not statistically significant.

**Table 6:** Sources of information and knowledge for primary care nurses

	**Age 30–49**	**Age 50 & over**	**All**	**Fisher 1 tail *p *=**
Books personal collection	75.0%	85.2%	78.9%	0.238
Journals personal collection	43.2%	51.9%	46.5%	0.320
Libraries	36.4%	29.6%	33.8%	0.376
Journals at work	75.0%	77.8%	76.1%	0.513
Electronic resources	29.5%	33.3%	31.0%	0.469
Books at work	68.2%	74.1%	70.4%	0.401
n=	44	27	71	

Nurses summarised their experiences in access information in a number of free text comments:

"don't know where to look", "don't know website address",

"vast amounts of irrelevant articles under same headings",

"I am not as good as I should be with CIHNAL, Medline",

"information not found or not relevant some difficulty sometimes refining the search for specific information"

"lack of training/unable to access information I am looking for quickly enough – quicker to look in a book"

"like trying to get a glass of water from Niagara Falls"

## Discussion

This study demonstrated that primary care nurses have high levels of access to IT and only a small minority have no access at all. Practice nurses, working within the surgery, all use the computerised medical record and nearly all feel confident to use it; over three-quarters have access to the Internet. However, community bases nurses in the second half of their careers despite receiving more training; have less access and are less confident about computers, especially use of the EPR, and prefer to use paper-based information resources. Across all ages and professions using libraries, reading journals and accessing on-line resources are minority activities. One to one learning in the workplace, and workshops were believed to be the best way to learn, with printed manuals and on-line tutorials of least value.

The implication of this study is that community nursing needs more silver surfers. Silver surfers are those over 50 years who enjoy using the Internet. The primary care nurses over 50, despite more training, are still looking for information on paper; using their computers less at work; and less confident in computer use. Market strategies are making the over 50 s the fastest expanding group of Internet users this decade [17,18]; but the benefits of this growth in the market have not as yet had sufficient impact on primary care nurses. The successful aspects of these strategies need to be replicated for community over 50 years if they are to become effective users of the EPR and access the ever increasing number of online information resources. Nurses have clear ideas about the sort of training they want; however their chosen options is the most labour intensive and potentially expensive for the health service to provide.

The small sample size and single locality of this study are its principal weaknesses. The former prevented any more detailed sub-group analysis, as the groups became too small. A further reminder may have improved the response rate.

This study shows that nurses have made enormous strides in acquiring IT skills and access to computers since earlier surveys [10-12]; although these surveys included a smaller proportion of practice nurses – the group that appears to have embraced information technology the most. However, the use of Internet sources of information has changed relatively little. The change strategies proposed by the nurses fit with much of the literature, even though most of this was written in the context of implementing evidence based guidelines. It is likely that lessons about changing one type of behaviour in medicine are likely to apply to others [19-21].

Further research is needed with a larger sample to see if some of the non-statistically significant trends seen in this data become significant in a larger sample drawn from a national sample. It is important for the NHS to understand what input is needed to raise the level of use of the EPR and online information; and how quickly this change can be achieved. What nurses believe to be most effective ways of changing behaviour, and promoting computer use needs to be tested.

## Conclusions

There is a consensus among primary care nurses of the sort of training they require to improve their computer use. A significant proportion of community nurses in the second half of their careers need this additional support if they are to achieve silver surfer status; and, have the skills needed to work in the new computerised NHS. Studies need to test whether providing the training wanted overcomes the barriers reported. Although primary care nurses have acquired more computer skills than previously reported there is no room for complacency – community nurses need the opportunity to develop into silver surfers.

## Abbreviations

CPHVA Community Practitioners and Health Visitors Association, a representative body of community nurses.

EPR Electronic patient record, synonymous with Computerised Medical Record.

IT Information Technology

NHS National Health Service. The UK's state funded health service.

PCEL Primary Care electronic Library, an on-line information resource for primary care.

PC personal computer

PCT Primary Care Trust – organises and commissions care for a locality of about 100,000 population.

RCN Royal College of Nursing

SNOMED CT Systematized Nomenclature of Medicine – Clinical Terms

SPSS Statistical Package for Social Sciences – a computerised statistical package.

## Competing interests

The author(s) declare that they have no competing interests.

## Authors' contributions

All authors conceived the study, and contributed to all aspects of the paper. The major contributions of each author is as follows: TC Analysed the data for the study, SB Recruited the nurses, sent out and collated the questionnaire, SdeL helped develop the questionnaire used in a earlier study and wrote the initial draft of the paper.

## Pre-publication history

The pre-publication history for this paper can be accessed here:



## Supplementary Material

Additional File 1 Community nurses access to and use of computers: questionnaire Click here for file
