# Supplementary material for: Community nursing needs more silver surfers: a questionnaire survey of primary care nurses' use of information technology
Source: BMC Nurs. 2004 Oct 7;3:4. doi: 10.1186/1472-6955-3-4 (PMC526210; doi:10.1186/1472-6955-3-4)
Supplement: Additional File 1 — Community nurses access to and use of computers: questionnaire [file 1472-6955-3-4-S1.doc]

Community nurses access to and use of computers

1. Which information resources do you use?
2. Please rate how confident you are when using the following:

|  | Not  confident | Quite confident | Confident | Very  confident | Never use | Don’t know |
| --- | --- | --- | --- | --- | --- | --- |
| A mouse | 1 | 2 | 3 | 4 |  |  |
| A keyboard | 1 | 2 | 3 | 4 |  |  |
| Word /other word processor | 1 | 2 | 3 | 4 |  |  |
| Excel/ other Spreadsheet | 1 | 2 | 3 | 4 |  |  |
| e-mail | 1 | 2 | 3 | 4 |  |  |
| Internet | 1 | 2 | 3 | 4 |  |  |
| Electronic library | 1 | 2 | 3 | 4 |  |  |
| Computerised patient medical records  (e.g. EMIS, Torex)  Comments: | 1 | 2 | 3 | 4 |  |  |

1. **Which of the following do you use to support your work?**

(You may select more than one box)

| Books | Personal collection |  | Work/dept collection |  |
| --- | --- | --- | --- | --- |
| Journals | Personal collection |  | Work/dept collection |  |
| Library |  |  |  |  |
| Colleagues |  |  |  |  |
| Electronic Resources  (e.g. Internet, electronic journals) |  |  |  |  |
| Other |  |  | (Please State) |  |

1. How often do you use a computer to support your work?

| At least daily |  |  |
| --- | --- | --- |
| At least weekly |  |  |
| At least monthly |  |  |
| Never |  |  |
| Don’t know |  |  |
| Other |  | (Please state) |

1. Which of the following do you prefer to use to support your work?

| Paper (e.g. books and journals) |  | (Please state why) |  |
| --- | --- | --- | --- |
| Computer (e.g. Internet and electronic journals) |  | (Please state why) |  |
| Both |  | (Please state why) |  |
| No preference |  | (Please state why) |  |

1. **Do you have access to the Internet?**

Yes 

No 

1. **If you have access to the Internet please indicate where:**

(You may select more than one box)

Work 

Home 

Other  Please state

1. **For what purpose do you use the Internet?**

|  | At least daily | At least weekly | At least monthly | Occasionally | Never |
| --- | --- | --- | --- | --- | --- |
| Literature Searching |  |  |  |  |  |
| Email |  |  |  |  |  |
| Drug Information |  |  |  |  |  |
| Information for patients |  |  |  |  |  |
| Other  (Please State): |  |  |  |  |  |

1. Do you regularly experience difficulty when trying to find the information you need from computers?

| Yes |  | Please state why |
| --- | --- | --- |
| No |  |  |
| Never use |  |  |

1. **Would you like to make more use of electronic resources?**

Yes 

No 

Please specify why in either case:

1. Have you ever received any training in the use of computers?

| Yes |  | Please list the subject areas in which training has been received: |
| --- | --- | --- |
| No |  |  |

1. **Would you like to receive training in the use of any of the following resources? (You may select more than one box)**

##### Databases  (Please State which ones)

Internet 

Other  (Please State)

1. What would be your preferred format for training?

|  | Least Desirable | Adequate | Desirable | Highly Desirable |
| --- | --- | --- | --- | --- |
| Printed manuals | 1 | 2 | 3 | 4 |
| Tutorial on the Internet | 1 | 2 | 3 | 4 |
| Lectures | 1 | 2 | 3 | 4 |
| Workshops | 1 | 2 | 3 | 4 |
| One to One | 1 | 2 | 3 | 4 |

## Please specify where you would like to receive this training:

| At work |  |
| --- | --- |
| At an education/teaching centre |  |
| Library |  |
| Home (self directed learning) |  |

**Please provide your details below:**

**(THIS INFORMATION WILL BE KEPT STRICTLY CONFIDENTIAL)**

**Gender**

Male 

Female 

# Which age group do you belong to?

Under 30 years 

30-39 years 

40-49 years 

50-59 years 

Over 60 years 

**Job Title**

District Nurse 

Health Visitor 

Practice Nurse 

Other  (Please State)

### **Place of Work**

###### Are you based at:

A Practice 

A Hospital 

Other  (Please State)

**Please return your completed questionnaire to: -**

**This questionnaire may be used in support of your work providing that The Primary Care Informatics Group at St George’s Hospital Medical School and KSSnet is fully acknowledged.**
